# Physiological response after translocation differs between source populations in a threatened mammal

**DOI:** 10.1098/rsos.230836

**Published:** 2023-12-13

**Authors:** Kelly S. Williams-Kelly, Laurence Berry, Kim Branch, Saul Cowen, Sean Garretson, Greg J. Holland, Rachel Ladd, Liberty Olds, Kelly Rayner, Colleen Sims, Leanne Van Der Weyde, Kylie A. Robert, Kerry V. Fanson

**Affiliations:** ^1^ School of Agriculture, Biomedicine and Environment, La Trobe University, Melbourne 3086, Victoria, Australia; ^2^ Research Centre for Future Landscapes, La Trobe University, Melbourne 3086, Victoria, Australia; ^3^ Department of Energy, Environment and Climate Action, Victorian Government, Melbourne 3000, Victoria, Australia; ^4^ Department of Biodiversity, Conservation and Attractions, Western Australian Government, Woodvale 6026, Western Australia, Australia; ^5^ School of Biological Sciences, University of Western Australia, Crawley 6009, Western Australia, Australia; ^6^ Australian Wildlife Conservancy, Buronga 2739, New South Wales, Australia; ^7^ Zoos South Australia, Adelaide 5000, South Australia, Australia; ^8^ Department of Environment and Water, South Australian Government, Adelaide 5000, South Australia, Australia

**Keywords:** conservation, faecal glucocorticoid metabolites, reintroduction, stress physiology, validation

## Abstract

Conservation translocations are an important tool in the prevention of species loss, but the translocation process is associated with numerous stressors. Non-invasively monitoring stress physiology via faecal glucocorticoid metabolites (FGMs) can provide valuable insights into factors impacting translocation success and how to mitigate negative impacts. After validating an assay to measure FGMs in greater stick-nest rats (*Leporillus conditor*), we examined whether translocation caused a predictable change in physiology. We compared longer-term (one to five months post-translocation) physiological responses across three source populations (remnant-wild, reintroduced-wild, captive-bred), and investigated effects of body condition and sex on FGMs. Notably, FGMs of the remnant-wild population did not significantly change post-translocation, while the reintroduced-wild population exhibited a significant decrease and the captive-bred population a significant increase. Individuals in lower body condition had the highest FGMs in both wild-type populations, whereas the captive-bred population showed the opposite relationship. There was no difference in FGMs between the sexes. Our work highlights that physiological responses after translocation may not be uniform and source population history is an important factor to be considered, emphasizing the need for novel ideas that facilitate successful adaptation. By better understanding how species and individuals respond to translocation, we can improve translocation outcomes.

## 1. Introduction

As extinction rates continue to increase, so too does the need to improve the outcomes of conservation actions for the species remaining. Translocation, or the human-assisted movement of animals from one location to another, is an important wildlife conservation tool and one increasingly being implemented in the race against extinction [1]. For example, translocation can be used to boost numbers in an existing population or reintroduce a species to part of its former range where local extinction has occurred, termed reintroduction [2]. However, translocation success rates are variable (see reviews, [3–5]). Translocation failure has largely been attributed to issues with predation, management and habitat quality [6], although the cause of failure is uncertain or unknown in many cases [3]. The process of reintroduction is often complex and involves several potentially stressful events for animals including capture, handling, health assessments, containment, transportation and release to a novel environment [7,8]. The physiological changes induced by translocation, a seldom measured metric, may be a contributing factor to success [7].

Monitoring animal physiology in a translocation can provide valuable insights into how animals experience translocation events, and reveal strategies to mitigate impacts of stress [9,10]. Determining what aspects of translocation are most stressful and how different cohorts respond could be used to improve animal welfare and assist the selection of individuals most suitable for translocation, enhancing success of future translocations. How an individual deals with stress is the result of a complex physiological system [11]. Glucocorticoids, which are hormones produced by the hypothalamic–pituitary–adrenal (HPA) axis, play an important role in stress responses [12]. Any stimulus that threatens the body's stable internal environment (homeostasis) can be considered a stressor, and glucocorticoids help coordinate an animal's response to the stressor by causing physiological and behavioural changes [13]. An adaptive response results in a short-term increase in glucocorticoids appropriate for the gravity of the stressor [8]. Many factors influence how an individual responds to a stressor, such as source population history, sex, age and body condition.

Translocated animals can be sourced from the wild or bred in captivity [14]. However, wild and captive animals have very different environmental and personal experiences [15]. Different source population types may subsequently differ in their physiological response after translocation. Persistent heightened stress can decrease reproduction and increase mortality [9,16]. These negative impacts are obviously not conducive to translocation success. Therefore, monitoring stress response is of interest to practitioners tasked with improving translocation outcomes [17]. Studies focusing on the immediate effects of translocation on glucocorticoids typically report an increase in concentrations [18,19]. However, much less is known about longer-term physiological changes, and even less when comparing responses of animals from different source populations [20]. Faecal hormone monitoring presents a relatively simple approach to provide such insights about stress physiology in wildlife.

Glucocorticoids are a widely used biomarker of stress and have traditionally been measured in blood. However, the compounding physiological impacts of capture, restraint, blood sample collection and post-sampling health monitoring make it unsuitable in many instances [21]. Faecal hormone analysis provides a non-invasive alternative, with faeces also much simpler to collect than blood [17]. Furthermore, blood sampling gives a snapshot of glucocorticoid levels at the specific time of sampling, while faecal samples contain average levels of hormone metabolites and provide information about the animal's experience over a period of time, dependent on defecation rates [22]. However, when the body metabolizes glucocorticoids they are broken into numerous metabolite structures and excreted, and every species, even those closely related, has a unique metabolism [23]. Therefore, the method being used to quantify the faecal metabolites must be validated to ensure that it is accurately measuring metabolites of the hormone of interest [24].

The goal of this study was to examine whether the process of translocation causes a predictable change in physiology. The literature suggests that translocations inevitably lead to a state of chronic stress [7], but few studies have compared post-translocation changes in physiology across multiple populations. In this study, we compared longer-term (one to five months post-release) physiological responses across three different translocations of greater stick-nest rats (GSNRs). Our focus was not on the (well-studied) acute stress response to capture and translocation, but instead on sustained physiological changes more indicative of acclimation. Specifically, we aimed to (i) biochemically and biologically validate an enzyme immunoassay to measure faecal glucocorticoid metabolites (FGMs) in GSNRs, (ii) describe how intrinsic factors such as body condition and sex affect FGM levels, and (iii) compare the physiological response (change in FGMs) of three different source populations after translocation. Notably, our study had the unique opportunity to include source populations with diverse natural histories: the last naturally occurring ‘remnant' wild population, a wild reintroduced population, and a captive-bred population. Under the current framework about the effect of translocations on physiology [7], we predicted that all populations would exhibit an increase in glucocorticoids. Our findings provide insight into how different populations respond after translocation and challenge existing frameworks about the physiological costs of translocations. Ultimately, we hope to apply this knowledge to improve future translocation outcomes.

## 2. Methods and materials

### 2.1. Study species

The greater stick-nest rat (*Leporillus conditor*) is an herbivorous rodent weighing 180–450 g (females and males similarly sized) that once occurred across semi-arid southern Australia. GSNRs occupy large communal nests constructed of sticks that are maintained over several generations, with breeding occurring non-seasonally [25]. Numbers rapidly declined after European settlement until the species became extinct on the mainland in the 1930s [25]. Today, the last naturally occurring GSNR population persists on the Franklin Islands off the coast of southern Australia [26]. GSNRs are one of two species in their genus, with lesser stick-nest rats (*Leporillus apicalus*) not officially observed since the 1930s and declared extinct in 2016 [27]. In efforts to increase numbers and restore historically extinct populations, GSNRs have been the subject of captive breeding programmes and several translocations, with varied success [28]. Identifying strategies to improve translocation outcomes is critical to the conservation of this species.

### 2.2. Source populations

**Figure 1.**
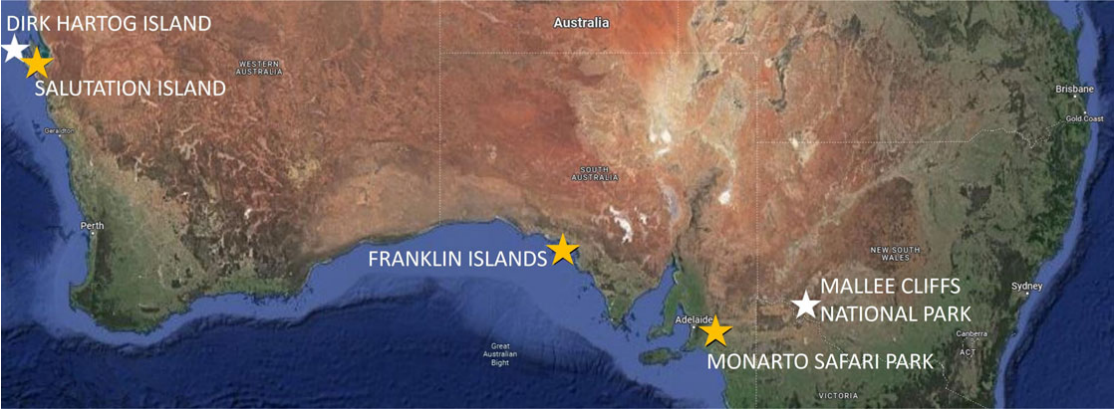
Greater stick-nest rat study site locations. Yellow star, pre-translocation ‘source' site; white star, post-translocation site. Original map imagery: Google © 2023, Data SIO, NOAA, U.S. Navy, NGA, GEBCO, Landsat/Copernicus, IBCAO; study site markers and labels manually added to image.

Samples were collected from GSNRs from three source populations (discussed below) at two time points (pre-translocation and post-translocation) (table 1). Spread across three states in Australia (figure 1), all pre-translocation and post-translocation locations have a semi-arid climate, with minimum temperatures ranging from −4.1 to 8.5°C, maximum temperatures from 40.1 to 45.1°C and total annual rainfall from 212.8 to 474.0 mm [29] during the study period (2021–2022).

**Table 1.** Overview of greater stick-nest rat populations in the translocation study.

sampling time point	population type (*N* = individuals; females, males; *n* = samples)	description	location and latitude, longitude
pre-translocation	captive-bred (*N* = 15; 9 F, 6 M; *n* = 19)	animals bred in captivity	Monarto Safari Park, South Australia 35.09° S, 139.16° E
pre-translocation	reintroduced-wild (*N* = 40; 25 F, 15 M; *n* = 51)	population reintroduced (via translocation) to island approximately 30 years ago	Salutation Island, Western Australia 26.54° S, 113.77° E
pre-translocation	remnant-wild (*N* = 20; 9 F, 11 M; *n* = 21)	last remaining naturally occurring population	Franklin Islands, South Australia 32.45° S, 133.65° E
post-translocation	captive-bred (*N* = 4; 2 F, 2 M, *n* = 6)	animals from Monarto Safari Park translocated in May 2021	Mallee Cliffs National Park, New South Wales 34.21° S, 142.62° E
post-translocation	reintroduced-wild (*N* = 8; 4 F, 4 M; *n* = 17).	animals from Salutation Island translocated in May 2021; animals from Franklin Islands translocated in May 2022	Dirk Hartog Island, Western Australia 25.78° S, 113.03° E
	remnant-wild (*N* = 3; 3 F, 0 M, *n* = 3)		

#### 2.2.1. Remnant-wild (Franklin Islands)

The last population of GSNRs to occur naturally is found on the Franklin Islands, two partially joined islands (East and West) in the Nuyts Archipelago Marine Park in South Australia [26]. All other living populations are thus derived from this remnant population [30]. The islands are a publicly prohibited area and no non-native mammal species are found there, only native GSNRs and Franklin Island southern brown bandicoots *Isoodon obesulus nautilus* [28]. The diet of GSNRs on the Franklin Islands consists of the leaves and fruits of succulent and semi-succulent plants, such as nitre bush (*Nitraria billardierei*) and saltbush (Chenopodiaceae) [25,28]. Predators on the islands include birds of prey and tiger snakes (*Notechis scutatus*).

#### 2.2.2. Reintroduced-wild (Salutation Island)

GSNRs were first released to Salutation Island in July 1990 with 40 animals translocated from a captive-bred colony at Monarto Safari Park [31]. The reintroduction of GSNRs to Salutation Island is one of the few successful translocations for the species to date, making it a valuable source for other translocations (for a review of translocation history in GSNRs, see [28]). Salutation Island is a small island in the southern end of Shark Bay, Western Australia, a UNESCO World Heritage listed area. While GSNRs were not officially known to previously occur on the island they were historically recorded throughout the Shark Bay area [32]. Saltbush and other semi-succulents comprise their diet. The species is the only mammal found on the island and are preyed upon by gwardar (western brown) snakes (*Pseudonaja mengdeni*) and birds of prey such as eastern barn owls (*Tyto javanica*).

#### 2.2.3. Captive-bred (Monarto Safari Park)

In 1985, the Monarto Fauna Complex, managed by the South Australian National Parks and Wildlife Service, began a captive breeding program of GSNRs using wild-caught animals sourced from the Franklin Islands. This captive colony was the source of GSNRs for several island and mainland translocations (including Salutation Island) and was maintained until 2003 when the last remaining individuals were transferred to other captive colonies [28]. The breeding program, now part of Monarto Safari Park, recommenced in 2019 with 30 animals sourced from West Franklin Island. The individuals translocated in 2021 and included in this study were primarily first-generation offspring, with some second-generation. Animals were housed in partly covered outdoor enclosures in groups of up to eight individuals and fed a diet of root and green vegetables, seeds, kangaroo pellets and saltbush.

### 2.3. Translocations

#### 2.3.1. Dirk Hartog Island

Dirk Hartog Island is the largest island in Shark Bay and was declared a National Park in 2009. It is open to the public and attracts fishing and camping tourists. Subfossil skeletal records show GSNRs once inhabited the island [32] but likely went extinct from habitat degradation as a result of livestock grazing and predation from feral cats in the nineteenth century [25,33]. The ‘Dirk Hartog Island National Park Ecological Restoration Project - Return to 1616' began in 2014, aiming to re-establish historical ecosystem processes and reintroduce several locally extinct native species, including GSNRs [33]. Feral cats, goats and sheep were eradicated from the island for this purpose by 2017. While non-native house mice (*Mus musculus*) are still found on the island, native mice species are abundant and a number of native mammal species have also been translocated as part of the ‘Return to 1616' program since 2017.

The first translocation of GSNRs to the island comprised reintroduced-wild GSNRs caught with Elliott and Sheffield traps on Salutation Island in May 2021 by the Western Australian Government Department of Biodiversity, Conservation and Attractions (DBCA) [34]. They were transported in individual wooden transport boxes via helicopter (approx. 30 min transit) and kept in these boxes in a quiet air-conditioned room until their release after dusk. Artificial nests were constructed using dead shrubs, sticks and foliage and GSNRs were released directly into these ‘protonests' with the transport boxes placed inside with the front door opened. They were then left to emerge voluntarily, with boxes remaining in nests as additional refuge. Available food resources for GSNRs include several succulent and semi-succulent shrubs, including saltbushes. Predators include raptors and reptiles, like Children's python (*Antaresia childreni*) and sand monitor (*Varanus gouldii*).

The second translocation of GSNRs to Dirk Hartog Island occurred in May 2022, coordinated again by DBCA, in which remnant-wild GSNRs were caught from the Franklin Islands using hand nets [35]. Their transit involved a short helicopter ride from the islands to the Australian mainland, followed by plane transport to Shark Bay and a second short helicopter ride to Dirk Hartog Island (approx. 9 h transit). The same translocation processes were enacted as the first translocation to the island, with placement of opened transport boxes into ‘protonests' after dusk.

#### 2.3.2. Mallee Cliffs National Park

Mallee Cliffs National Park was reserved in 1977 and today is managed by Australian Wildlife Conservancy (AWC) on behalf of New South Wales National Parks and Wildlife Service [36]. In 2018, a 9640-hectare feral predator-proof fence was constructed around a section of the park, with feral cats and foxes eradicated from within this area. Non-native house mice and European rabbits (*Oryctolagus cuniculus*) still occur inside the fence, although rabbit numbers are very low and are controlled by targeted shooting. Several translocations of regionally extinct mammal species have occurred inside the fence. The last official observation of GSNRs in New South Wales was recorded in 1862 near the Murray River and Darling River junction [37], about 10 km from Mallee Cliffs National Park.

GSNRs were first reintroduced to a 480-hectare fenced area of the park in September 2020. Samples from GSNRs included in the current study were captive-bred at Monarto Safari Park and translocated to the adjacent, 9640-hectare fenced area of the park in May 2021. They were transported in individual wooden boxes via car (approx. 6 h transit) and, similarly to other translocations, kept in an air-conditioned room until their release after dusk. Release sites were selected based on the availability of food plants and cover provided by *Acacia ulicifolia*. Artificial nests were constructed using sticks and large plastic tubing inside soft release pens made from plastic dampcourse sheeting, which were then opened after 48 h. These pens were used to limit over-dispersion and facilitate survival monitoring efforts post-release. Unlike the other two translocations, upon release of GSNRs transport boxes were removed. The Park contains several succulent and semi-succulent shrubs for GSNRs to eat, including saltbushes, and is home to native predators including snakes, sand monitors and birds of prey.

### 2.4. Faecal sample collection

GSNRs often defecate during capture and handling, allowing faecal samples to be collected non-invasively. Fresh faecal samples were collected from several collection points, including behavioural testing arenas (*n* = 22 samples, captive-bred GSNRs underwent brief individual behavioural tests in an arena as part of a larger study a month prior to translocation), from traps (*n* = 95 samples) and from handling bags (*n* = 51 samples). Pre-translocation samples for both wild populations were collected at the time of capture for the translocation event and the captive-bred population samples were collected one month prior to translocation. Post-translocation samples were then collected during monitoring sessions after one and four months for the captive-bred population, one and five months for the reintroduced-wild population and after four months for the remnant-wild population. Collected faecal pellets were placed in 1.5 ml Eppendorf tubes labelled with the date, the individual's passive integrated transponder (PIT) number and collection point. Samples were then stored at −20°C until steroid extraction.

### 2.5. Enzyme immunoassay validation

Biochemical validation was achieved for each assay by demonstrating parallelism between serial dilutions of faecal extracts (pooled samples from 12 individuals, 6 males and 6 females) and the standard curve. For the biological validation, we measured changes in FGMs resulting from repeated capture and handling, a known stressful event [38,39]. Five wild-caught individuals (three non-lactating and non-pregnant females, two males) from Salutation Island and Mallee Cliffs National Park were incidentally recaptured one to two nights after their initial capture, so we compared first capture FGMs with recapture FGMs for each individual. During the capture surveys, baited Elliott and/or Sheffield traps were set in the early evening and then checked within 3 h from sunrise, with the faeces deposited by each captured GSNR collected. To determine the most suitable enzyme immunoassay for measuring FGMs for the species, we compared the performance of three different assays, referred to as AA-F, Cs6 and 37e. These assays were selected as each targets a different glucocorticoid hormone or metabolite, and has been previously validated in rodents or other mammal species (see below for assay procedures and species' validation references).

### 2.6. Steroid extraction

Steroids were extracted from the faeces by adding 1 ml of 80% ethanol to 0.04 ± 0.001 g of wet faecal material in a 5 ml polypropylene tube. Samples containing several pellets had sub-samples taken from each pellet to avoid bias from any single pellet. The tubes were vortexed and shaken on an orbital shaker overnight. Samples were vortexed again and then centrifuged for 5 min at 2380 RCF. The supernatant was decanted into a new 1.5 ml tube and stored at −20°C until analysis.

### 2.7. Assay procedure

Three double-antibody enzyme immunoassays were first trialled with a pooled subset of samples to determine the most appropriate assay to quantify GSNR FGMs (assay validation described above). The first assay was directed at cortisol and hereafter referred to as AA-F (Arbor Assays, Michigan, USA, Cortisol ISWE mini-kit catalogue #ISWE002). The second was directed at corticosterone and referred to as Cs6 (produced at University of California Davis, Davis, USA; antibody and corresponding horseradish peroxidase (HRP) conjugate obtained from Smithsonian Conservation Biology Institute, Virginia, USA, first described by Watson *et al*. [40]. The third assay was termed 37e and was directed at 5a-pregnane-3ß,11ß,21-triol-20-one (University of Veterinary Medicine, Vienna, Austria, described by Touma *et al*. [41]). For each assay, methods were like those previously described, with small variations explained below. Metabolite concentrations are expressed as ng g^−1^ of wet faecal weight.

For the AA-F assay, plates were washed four times with 300 µl wash solution and loaded with 50 µl of buffer, standard, control or sample. Next, 25 µl of cortisol conjugate (1 : 50) was loaded, followed by 25 µl cortisol antibody (1 : 50). Plates were shaken for 2 h at room temperature then washed four times. Then 100 µl tetramethylbenzidine (TMB) solution was loaded and the plates were incubated on the shaker. After 1 h incubating the reaction was stopped with 50 µl sulfuric acid (2 M) and the optical density was read (450 nm measuring filter, 620 nm reference) on a SPECTROstar Nano Plate reader (BMG LABTECH). Inter-assay coefficients of variation (CVs) for low (approx. 25% binding) and high (approx. 75% binding) controls were 5.26% and 11.61%, respectively (*n* = 7 plates). Intra-assay CVs for low and high controls were 3.23% and 1.91%, respectively (*n* = 12).

For the Cs6 assay, plates were washed five times with 200 µl wash solution and loaded with 50 µl of buffer, standard, control or sample. Then 50 µl corticosterone HRP solution (1 : 80 000) was loaded, followed by 50 µl corticosterone antibody (1:100 000). Plates were shaken for 2 h at room temperature, washed five times and loaded with 150 µl 2,2′-azino-bis(3-ethylbenzothiazoline-6-sulfonic acid)solution. After incubating on the shaker for 45 min, the optical density was read (405 nm measuring filter, 490 nm reference). Intra-assay CVs for low and high controls were 4.17% and 12.02%, respectively (*n* = 16).

For the 37e assay, plates were washed three times with 300 µl washing solution and loaded with 50 µl of buffer, standard, control or sample. Next, 50 µl of corresponding biotin-label (1 : 40 000) was loaded, then 50 µl antibody (1:30 000). Plates were shaken overnight at 4°C. The next day, plates were washed four times and 150 µl streptavidin–peroxidase solution was loaded. After 45 min of shaken incubation at 4°C, plates were then washed and 150 µl TMB solution was loaded. Plates were incubated on the shaker at room temperature for one hour. The reaction was stopped with 50 µl sulfuric acid (2 M) and the optical density was read (450 nm measuring filter, 620 nm reference). Intra-assay CVs for low and high controls were 3.54% and 2.76%, respectively (*n* = 12).

### 2.8. Body condition and sex

Each time a GSNR was caught, it was placed in a calico handling bag and scanned for a PIT for identification (where no PIT was found, one was administered under the loose skin near the back of the neck). Each animal was then weighed to the nearest 5 g with a Pesola spring scale, sexed based on the presence of testes (males) or mammary glands (females), and had its pes (hind foot) length measured to the nearest millimetre. Only samples from adult GSNRs (greater than 100 g body weight) were included in the study as juveniles were not translocated. Samples from lactating and pregnant females were excluded from analysis. Body condition score was then calculated by using the residuals from an ordinary least-squares regression of pes length and body weight, with females and males calculated separately [42]. An individual's score represents the vertical distance between the residual data point and the regression line, with greater numbers (either negative or positive) demonstrating greater distance from the line. Therefore, a highly negative body condition score (e.g. −100) indicates this individual's weight was much lower than predicted for a GSNR with its pes length.

### 2.9. Statistical analyses

All data were analysed using R 4.1.1 [43]. FGM concentrations were log transformed to meet model assumptions of a normal distribution and homoscedasticity. Statistical significance was set at *α* = 0.05.

#### 2.9.1. Enzyme immunoassay validation

We performed paired *t*-tests for each of the three assays comparing first capture and recapture FGMs. Changes in FGM concentration were then described as a fold increase or decrease.

#### 2.9.2. Body condition and sex

To investigate the impact of body condition and sex on FGMs, we ran a linear mixed model (LMM) using the R program packages lme4 (version 1.1-25 [44] and lmerTest [45]. The package ggplot2 [46] was also used to visualize and present results. FGM concentration was modelled as a function of sex and the interaction between body condition and source population (remnant-wild, reintroduced-wild, captive-bred). Pre- and post-translocation samples were included to maximize sample sizes. Animal ID was included as a random effect due to repeated sampling of some individuals. The emmeans package [47] was then used to test pair-wise comparisons of slopes (FGM×body condition) between populations.

#### 2.9.3. Source population and translocation

To examine how different populations physiologically responded after translocation, we used an LMM. FGM concentration was modelled as a function of the interaction between source population (remnant-wild, reintroduced-wild, captive-bred) and translocation stage (pre-translocation, post-translocation), with animal ID included as a random effect to account for repeated sampling. We then performed a post-hoc pair-wise comparison of mean FGMs within each source population to examine how FGMs changed after translocation. We were also interested in comparing pre-translocation and post-translocation FGMs between the source populations so another pair-wise comparison was conducted. Sex and body condition were included in the initial LMM, however, both variables were not significant so were removed from the final model. Post-translocation samples were collected at two time points for the captive-bred and reintroduced-wild population: one-month post-release and four (captive-bred population) or five (reintroduced-wild population) months post-release. Within each population there was no significant difference in FGMs between the two time points so they were grouped.

## 3. Results

### 3.1. Assay validation

For the biochemical validation, all three assays demonstrated parallelism between serially diluted extracts and the standard curve. We also demonstrated significant recovery of exogenous hormone added to faecal extracts.

**Figure 2.**
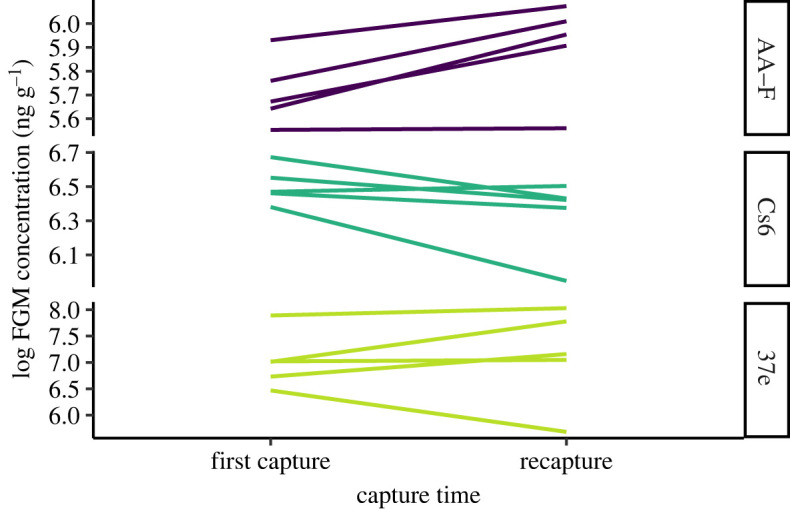
Change in faecal glucocorticoid metabolite (FGM) concentration between first capture and recapture of GSNRs (*N* = 5). Performance of three different enzyme immunoassays (AA-F, Cs6, 37e) was compared.

For the biological validation, only AA-F detected a significant increase in FGM concentrations at recapture (*t*_4_ = −3.5714, *p* = 0.023) (figure 2). AA-F also demonstrated the most consistency, with all five individuals showing an increase in FGMs (ranging from 1.01 to 1.37-fold). Neither Cs6 nor 37e detected a significant change between the two capture events (Cs6: *t*_4_ = 2.175, *p* = 0.095; 37e: *t*_4_ = −0.444, *p* = 0.680). For 37e, four out of five individuals had higher FGMs at recapture, while one individual had lower levels (average of 1.26-fold increase). Cs6 contrasted with the other two assays, showing a slight decrease in FGMs for four out of five individuals at recapture (average of 0.85-fold decrease). As AA-F was the most sensitive to changes in FGMs across recapture events and exhibited consistent directional results for all individuals, we considered it the most suitable assay for measuring FGMs in GSNRs and subsequently used it for all further analyses.

### 3.2. Body condition and sex

**Figure 3.**
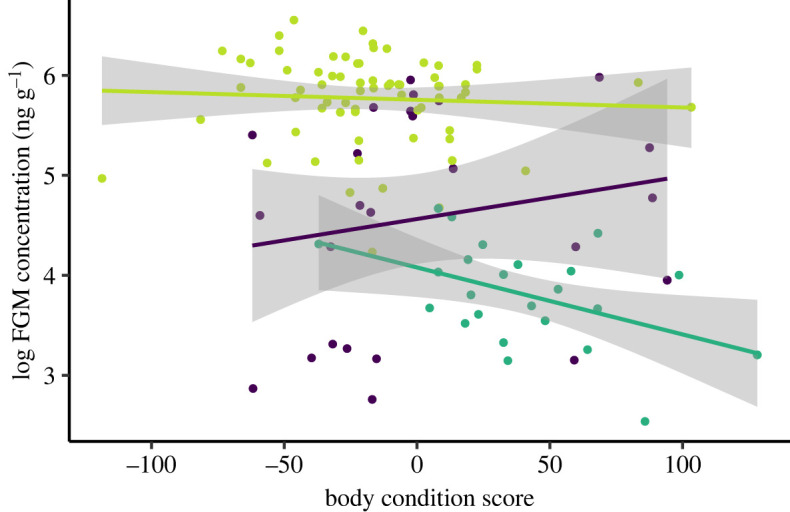
Relationship between body condition score and FGM concentration in GSNRs. Points represent individual animals (pre- and post-translocation samples). Dark green, remnant-wild population (slope = −0.007); light green, reintroduced-wild population (slope = −0.001); purple, captive-bred population (slope = 0.005). Grey shading indicates 95% confidence intervals.

The interaction between body condition and source population significantly affected FGMs (*F*_2, 93.067_ = 3.273, *p* = 0.042). The directional relationship between body condition and FGM concentration was then different among source populations (figure 3). Both wild populations showed a negative relationship between FGMs and body condition (i.e. individuals with lower body condition scores had higher FGMs). The captive-bred population showed the opposite relationship, in which individuals with higher scores had higher FGMs. Pair-wise comparisons showed the slopes for the remnant-wild population and captive-bred population were significantly different (*t*_103.7_ = −2.526, *p* = 0.035), whereas the slope for the reintroduced-wild population was not significantly different to either of the other two populations (remnant-wild: *t*_104.5_ = −1.427, *p* = 0.331; captive-bred: *t*_90.3_ = 1.453, *p* = 0.319). Sex did not significantly impact FGM levels (*F*_1, 69.409_ = 0.192, *p* = 0.663).

### 3.3. Translocation

**Figure 4.**
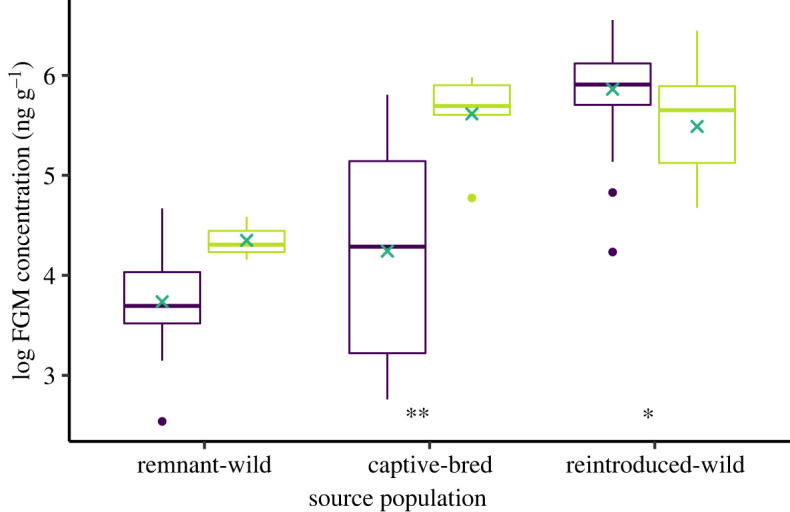
Changes in FGM concentrations of GSNRs pre- and post-translocation. Purple = pre-translocation ‘source' concentrations; light green = post-translocation concentrations. Within each box: dark green cross, mean; middle bar, median; bottom bar, first quartile; top bar, third quartile; bottom and top whiskers, minimum and maximum, respectively; coloured points, outliers. Statistical significance between pre- and post-translocation FGMs within each source population: *p < 0.05; **p < 0.0001.

Pre-translocation, the remnant-wild population had the lowest average FGM concentrations (46.6 ± 23.1 ng g^−1^), followed by the captive-bred population (108.1 ± 24.3 ng g^−1^), then the reintroduced-wild population with the highest (374.8 ± 14.9 ng g^−1^). Post-hoc pair-wise comparisons indicated pre-translocation average FGMs of the remnant-wild and captive-bred population were not different (*t*_103_ = −1.875, *p* = 0.151), while reintroduced-wild population average FGMs were significantly higher (remnant-wild: *t*_103_ = −11.804, *p* ≤ 0.0001; captive-bred: *t*_103_ = −10.492, *p* ≤ 0.001). Post-translocation, the response level and directional change in FGMs was not consistent across the three populations (figure 4). Average FGMs of the remnant-wild population increased slightly but not significantly (*t*_103_= 01.322, *p* = 0.189), while they significantly increased for the captive-bred population (*t*_103_ = −5.152, *p* ≤ 0.0001) and significantly decreased for the reintroduced-wild population (*t*_103_ = 2.289, *p* = 0.024). Post-translocation average FGM concentrations of the captive-bred population and reintroduced-wild population were then similar (*t*_103_ = 0,466, *p* = 0.887), and both were significantly higher than concentrations of the remnant-wild population (*t*_103_ = −3.145, *p* = 0.006; *t*_103_ = −3.198, *p* = 0.005, respectively).

## 4. Discussion

Translocations play an important part in species conservation. However, they are associated with several potentially stressful events that can have adverse physiological consequences, thereby threatening translocation success [48]. To examine whether animals show a consistent response to translocation, we monitored sustained physiological changes across three different translocations of GSNRs. In contrast to the current framework [7], we found that there was not a uniform increase in FGMs across populations. Only one population showed the predicted increase in FGMs, whereas one showed no change, and the third population showed a significant decrease in FGMs. This challenges the existing framework and highlights exciting opportunities to capitalize on these differences in order to improve translocation outcomes. By improving our understanding of how species and individuals respond after translocation, we can improve both translocation and animal welfare outcomes [7,50].

### 4.1. Validation

We compared the performance of three different assays for monitoring adrenal activity in GSNRs. All three of the assays were biochemically validated by demonstrating parallelism with the standard curve and exogenous hormone recovery. The biological validation showed that AA-F was overall the most suitable as it provided the most consistent results between individuals and a significant difference in FGMs between first capture and recapture. The GSNRs' level of response to capture and handling (1.01–1.37-fold increase) was similar to that of another Australian mammal, the bridled nailtail wallaby *Onychogalea fraenata* [51]. This response is relatively low compared with other studies, for example, a threefold increase was observed in wood mice *Apodemus sylvaticus* [52] and an eightfold increase in grey mouse lemurs *Microcebus murinu* [38]. However, it is important to note that FGM measurements taken at recapture in our study were likely not representative of the GSNRs' maximal responses. Repeated sampling over several days after a known stress event would have enabled us to make inferences about peak FGM levels and estimate the species' excretion lag time [23,53], but these short-term stress indicators were unnecessary for our longer-term study goals. Some GSNRs displayed minimal change in FGMs at recapture, indicating they did not find capture as disruptive as other individuals. High levels of individual variation in stress responses are common across species [38]. For example, a study on brushtail possums (*Trichosurus vulpecula*) found that some individuals showed no FGM response within five days of a potential stressor (entering rehabilitation), while others responded almost immediately [54]. Our results reinforce the need for assay validation and comparisons of assay performance to ensure biologically relevant data are being collected [39].

### 4.2. Body condition and sex

Source population type significantly impacted the association between FGMs and body condition, with both wild populations having a negative relationship with FGMs and the captive-bred population demonstrating a positive relationship. This indicates that different factors in the wild and in captivity were influencing FGM concentrations of GSNRs, which is not surprising given their different environments. Several factors can impact body condition and FGMs, some of which are mutually exclusive. In the wild, animals in poorer condition may have higher FGMs due to malnourishment or illness/disease supressing immune function and heightening HPA activity [13]. Woylies (*Bettongia penicillata*) another threatened Australian mammal, sourced from the wild similarly showed a negative association between body condition and FGMs after translocation [55]. Conversely, in captivity starvation and illness are largely controlled, minimizing their effects on body condition. Thus, for individuals in better body condition, increased social competition or lack of mental stimulation in captive settings could be cause for higher FGMs [15]. It is also worth noting there is presumably a point in which a high body condition score is no longer beneficial (i.e. obesity), but several different measures of health would be needed to identify this threshold. Body condition was also largely confounded with source population in this study which could have contributed to the differences between populations.

We found no difference in FGMs between female and male GSNRs, which is comparable to other rodent species such as the fawn-footed mosaic-tailed rat *Melomys cervinipes* [56] and Eurasian red squirrel *Sciurus vulgaris* [57]. While our study did not look at reproductive condition (such as the different stages of oestrous), its impacts on FGMs are well documented [57–59] and warrant further investigation in this species.

### 4.3. Translocation

Before translocation, the reintroduced-wild GSNR population had the highest overall FGM concentrations, followed by similar average levels between the captive animals and the remnant-wild population. One hypothesis for this disparity between the two wild-type populations is that the reintroduced-wild population had high population density on Salutation Island causing resource constraints and subsequent high stress levels. The population on Salutation Island is typically less than 500 individuals [28], while estimated densities at the time of pre-translocation sampling were substantially higher at 2230–3438 individuals [60]. In comparison, the remnant-wild population on the Franklin Islands had prime conditions at the time of sampling, with an abundance of food and shelter resources, and resources of the captive-bred population were largely controlled. This aligns with similar habitat impacts on physiology in other species. Yucatan spider monkeys (*Ateles geoffroyi yucatanensis*) in fragmented forests had higher FGMs than those in conserved forests, with the authors hypothesizing dietary and behavioural stress as possible drivers [61]. Furthermore, a study on captive and wild Siberian tigers (*Panthera tigris altaica*) found that wild tigers had the highest FGMs and they occurred during months of deep snow cover when food was most scarce, necessitating greater energy mobilization and increased HPA activity [62]. We hypothesize the reintroduced-wild GSNRs were similarly experiencing high physiological stress because of resource limitations.

Following translocation, the physiological response across the three populations was not consistent and therefore not in line with our prediction. The captive-bred population showed a significant increase in FGMs in the months following their translocation to Mallee Cliffs National Park. This is somewhat unsurprising given that this group was naive to life in the wild and therefore experiencing a plethora of novel stressors, including finding food, shelter and avoiding predators [7]. Captive-bred Persian fallow deer (*Dama mesopotamica*) released to the wild exhibited similar increases in FGMs [63]. The majority of these GSNRs were first-generation captive-bred (i.e. their parents were wild-caught), so further research could investigate how the number of generations in captivity impacts adaptation after release. For example, first-generation Atlantic salmon had half the reproductive success of wild-born fish [64], which could evidently impact long-term translocation success.

Faecal glucocorticoid metabolite concentrations of the remnant-wild GSNR population did not significantly change, indicating similar HPA activity before and after translocation and thus acclimating well. We note that small post-translocation sample size (*n* = 3) may have limited the statistical power. However, the remnant-wild GSNRs were experienced with the stressors associated with surviving in the wild and environmental conditions at the release site were good, which supports our result. A translocation study in greater rheas (*Rhea americana*) comparing two different source types yielded a similar pattern [65]. Birds bred in small pens with all food provided (intensive captivity) and birds bred in large paddocks with natural grazing and limited supplementary feeding (semi-extensive captivity) both had increased FGMs 60 days post-release. However, the physiological response was greater in the intensive captivity group (3.2-fold increase for females, 4.5-fold increase for males) compared to the semi-extensive group (2-fold for females, 2.6-fold for males) [65].

Conversely, the reintroduced-wild population demonstrated a significant decline in FGMs in the months following translocation to Dirk Hartog Island. As they too were familiar to the normal stressors of the wild, the large reduction in FGMs suggests they were less stressed in the new habitat, supporting our theory that the population was in distress pre-translocation on Salutation Island. Once released onto Dirk Hartog Island where high population density impacts were alleviated and resources were abundant, HPA activity and glucocorticoid secretion could begin to decrease from the population's potentially chronically high levels [9]. Long-term monitoring for all translocated populations is ongoing, so it remains to be seen if post-translocation FGM concentrations of the reintroduced-wild population will continue to decline to similar levels to those of the remnant-wild population. In Grevy's zebra (*Equus grevyi*), FGMs had returned to pre-capture levels four to six months post-translocation [66]. Conversely, a study on African elephant (*Loxodonta africana*) translocations found that FGM levels decreased very slowly, just 10% lower after 10 years and 40% lower after 24 years [20], indicating that sustained physiological impacts need to be monitored post-release.

There were several factors that may have contributed to the differences in physiological response. These factors highlight potential directions for future experimental studies, now that we know populations can respond to translocations in very different ways. Environmental differences between the two translocation sites may have contributed to FGM differences between the wild-type populations and the captive-bred population post-translocation. For example, water voles (*Arvicola amphibius*) at higher quality sites had lower FGM concentrations [67]. While both were assessed as being suitable, Dirk Hartog Island may have been a higher quality release site for GSNRs at the time of translocation than Mallee Cliffs National Park. Nonetheless, both were supplemented with artificial refuges, had appropriate food resources and similar native predators. Further research is thus needed to determine why the populations responded differently and which (if any) site conditions were more suitable for GSNRs. Season may have also impacted results and has been shown to affect FGMs in several mammalian species (African elephant [20], arctic ground squirrel [68], eastern bettong [69], Eurasian red squirrel [57], Siberian tiger [62], yellow-bellied marmot [58]. However, all translocations occurred in May (Australia's autumn) and both release sites had semi-arid climates with similar climatic conditions (rainfall and temperature data from [29]. Given that each GSNR population showed a different response after translocation, seasonal and environmental effects require further investigation.

Capture technique, transport mode and transit time can also affect stress levels [8]. Pre-translocation capture technique was largely confounded by source population, and it is not known which of the techniques (brief but intense hand/net capture or capture in a baited trap for several hours) is more stressful for GSNRs. Transport mode was also confounded by translocation site as both wild-type populations released to Dirk Hartog Island were transported by air, compared to road travel for the captive-bred GSNRs released to Mallee Cliffs National Park. Longer time in transit to the translocation site would presumably be more stressful, but regardless of transit time all populations were contained until they were released after dusk, reducing time-based effects. This lack of effect is supported by the remnant-wild population having the smallest physiological response but the longest transit duration. Importantly, it is unlikely that differences in capture and transport methods would still be impacting FGMs several months after translocation as they are largely short-term stressors.

The presence of resident GSNRs already at the translocation site could have impacted how newly translocated GSNRs acclimated to the environment. The initial translocation sites at Mallee Cliffs National Park and Dirk Hartog Island did not have resident GSNRs, meaning both the captive-bred source population and reintroduced-wild population needed to establish themselves post-release. Conversely, the remnant-wild population was released on Dirk Hartog Island nearby (approx. 1 km) to where the reintroduced-wild population had been released the year prior. The presence of an already established population may have positively influenced the newly translocated population's ability to survive and settle [49,70], subsequently contributing to a smaller FGM response. There was also some capacity for GSNRs from the 2020 release in the smaller fenced area of Mallee Cliffs National Park to climb over the fence into the larger 2021 release area, although their numbers (and thus effects) would have been minimal. Finally, post-translocation sample sizes for all source populations were small due to low capture rates of GSNRs after release (and not all captured individuals produced scat samples), so our measures of FGMs from limited individuals may not be representative of the population. Despite frequent visual observations of GSNRs during monitoring providing evidence of their continued persistence, low trappability was exacerbated by more abundant species occupying traps. Low recapture rates after release can be a common issue in translocations, despite significant sampling efforts.

This study revealed that chronic stress is not an inevitable component of translocations [7], but rather that populations can respond in very different ways. Future studies should aim to pinpoint the factors that contribute to different responses in order to maximize reintroduction outcomes. Our work highlights the need for further study into factors affecting stress physiology in wildlife and the physiological changes after translocation. Wild-to-wild translocations appear to minimize stress as the wild-type GSNR populations showed smaller FGM differentials, lending support to sourcing animals from wild environments. However, sourcing from the wild is not always feasible, such as from populations with limited numbers. How animals adapt after a translocation ultimately affect its success. Therefore, we must continue to work toward increasing successful acclimation post-translocation, such as choosing resilient individuals, minimizing capture and transport stress, selecting high-quality release sites, supplementing food and habitat resources and reducing predation pressures. As the movement of animals is increasingly being used as a conservation tool, scientists should continue to learn how to improve the likelihood of translocated animals successfully establishing a sustainable population in their new habitat.

## Data Availability

Supporting data are available online at Figshare repository doi:10.26181/23484743 [71].
